# Serum Beta-Human Chorionic Gonadotropin (β-hCG) Trends and Their Association With Abdominal Wound Healing After Cesarean Section: An Observational Study

**DOI:** 10.7759/cureus.117049

**Published:** 2026-09-28

**Authors:** Antima Vishwakarma, Kirti Kaithwas, Lavina Chaubey, Mamta Singh, Arvind Pratap Singh, T. B. Singh

**Affiliations:** 1 Department of Obstetrics and Gynecology, Institute of Medical Sciences, Banaras Hindu University, Varanasi, IND; 2 Department of General Surgery, Institute of Medical Sciences, Banaras Hindu University, Varanasi, IND; 3 Department of Community Medicine, Institute of Medical Sciences, Banaras Hindu University, Varanasi, IND

**Keywords:** abdominal wound healing, cesarean section, postoperative recovery, serum β-hcg, wound outcome

## Abstract

Background and objective

Beta-human chorionic gonadotropin (β-hCG) is an established biomarker in pregnancy assessment and in the management of trophoblastic and germ cell disorders. Beyond its classical endocrine role, β-hCG has biological relevance to inflammation, angiogenesis, endothelial function, immune regulation, and tissue remodeling, all of which are central to wound repair. The relationship between postoperative β-hCG decline and abdominal wound healing after cesarean section remains insufficiently characterized. The objective of this study was to evaluate the association between serial postoperative serum β-hCG trends and abdominal wound healing following cesarean section.

Methods

This hospital-based observational analytical study included 436 women undergoing elective or emergency lower-segment cesarean section. Baseline demographic, obstetric, and perioperative variables were recorded. Serum β-hCG levels were measured preoperatively, immediately postoperatively, and on postoperative days (PODs) 2, 3, 4, 5, 7, 14, and 21. Post-cesarean abdominal wound outcomes were clinically assessed and categorized as healthy or unhealthy. Comparative analysis and receiver operating characteristic curve analysis were performed.

Results

Mean β-hCG declined from 15,881.99 IU/L preoperatively to 143.13 IU/L on POD 21. Preoperative and immediate postoperative β-hCG levels did not differ significantly between wound groups. From POD 2 onward, women with unhealthy wounds had significantly higher β-hCG levels than those with healthy wounds (p < 0.001). POD 7 β-hCG showed excellent discrimination, with an area under the curve of 0.901. A cutoff of 1884 IU/L yielded 82.6% sensitivity and 81.2% specificity.

Conclusions

Slower postoperative β-hCG decline was associated with impaired abdominal wound healing after cesarean section. POD 7 β-hCG may serve as an adjunctive biomarker for early risk stratification.

## Introduction

Pregnancy, childbirth, and the early postpartum period represent major physiological and clinical transitions with important implications for maternal recovery, neonatal care, and family well-being [1]. Delivery can occur vaginally or by cesarean section, which is a procedure that involves an incision in the abdomen and uterus [2]. Cesarean section is an important obstetric procedure when vaginal delivery may compromise maternal or fetal well-being [3]. The growing prevalence of cesarean section in various health care institutions has led to a larger group of women at risk of experiencing postoperative wound healing complications following abdominal surgery, and thus wound healing has become a clinically relevant aspect of modern obstetric practice [4]. Risks to the mother include anesthetic complications, bleeding, infection, thromboembolism, injury to adjacent organs, and morbidity from subsequent abdominal surgery. Cesarean delivery increases the risk of placenta previa, placenta accreta spectrum, dense adhesions, difficult dissection, and hysterectomy, which in turn increases the risk of repeat cesarean delivery [5]. Wound complications after cesarean section can cause delayed mobilization, a longer hospital stay, a higher risk of readmission, increased health care costs, and maternal inability to care for their newborn in the early puerperium [6]. Early recognition of impaired wound healing is especially important in obstetric units, particularly in centers with high-volume deliveries, both elective and emergency [2].

Postsurgical wound healing is a complex biological process that includes hemostasis, inflammation, cellular proliferation, angiogenesis, collagen synthesis, epithelialization, and tissue remodeling [7]. In order for healing to occur, there must be a coordinated interaction between the integrity of the blood vessels, the immune system, the formation of the extracellular matrix, oxygen delivery, and local tissue perfusion [8]. Impaired healing can result in surgical site infection, gaping wounds, serous or purulent wound drainage, or partial or total failure of wound closure [4]. Dehiscence is often clinically apparent during the first postoperative week, when wound tensile strength is low and the incision is exposed to infectious, mechanical, metabolic, and inflammatory stress factors [9]. There are several factors affecting the healing of the abdominal wound following cesarean section, both related to the mother and to the procedure [10]. Patient-related factors include age, BMI, anemia, nutritional status, diabetes mellitus, hypertension, hypothyroidism, immune status, previous surgery, and parity [11]. Procedure-related factors include emergency surgery, the duration of the procedure, blood transfusion, intraoperative complications, the type of closure, placement of a drain, wound care after surgery, and antibiotic prophylaxis [12]. Malnutrition and hypoalbuminemia can lead to decreased fibroblast proliferation, collagen production, angiogenesis, and immune function [13]. Microvascular dysfunction, delayed leukocyte activity, oxidative stress, and changes in extracellular matrix deposition are all ways that diabetes mellitus can affect tissue repair [14]. Obesity can be associated with increased wound tension, poor tissue perfusion, changes in inflammatory activity, and increased susceptibility to surgical site infection [7].

Clinical assessment remains the main way to assess post-cesarean wounds. The typical assessment targets erythema, edema, discharge, tenderness, gaping, infection, delayed epithelialization, and dehiscence [15]. Signs and symptoms may not become apparent until after biological impairment has occurred, which may limit the ability to establish clinical risk stratification [9]. A measurable biomarker of biological recovery after surgery could allow for more intensive monitoring and prompt action in women at higher risk of poor wound healing outcomes [16]. The highly dynamic endocrine, immune, vascular, and metabolic changes experienced during pregnancy help support the developing fetus and alter the way that tissues respond to injury [11]. Cesarean section is performed in this pregnancy-specific physiological environment, which differs from the physiological environment of nonobstetric abdominal surgery, and so post-cesarean wound repair is not a typical surgical wound healing process [14]. hCG is a pregnancy-associated glycoprotein hormone, and its beta subunit is highly utilized in clinical practice for pregnancy assessment and for monitoring trophoblastic and germ cell disorders. Serum beta-human chorionic gonadotropin (β-hCG) falls gradually after delivery, with a measurable postpartum clearance pattern, due to the discontinuation of placental hormone production [5].

In traditional use, β-hCG has been known to be responsible for maintaining corpus luteum function and the production of progesterone in early pregnancy [13]. Other biological activities of β-hCG include immunomodulatory, angiogenic, endothelial, inflammatory, and tissue-remodeling effects [16]. These pathways share common mechanisms with some important processes in wound repair, which indicates a likely correlation between postpartum β-hCG dynamics and abdominal wound healing after cesarean section [6]. A less rapid decrease in serum β-hCG could either reflect a change in the endocrine resolution of the postpartum period or a delay in the systemic recovery of the postnatal organism [10,17]. The available information in the literature regarding the morbidity of cesarean sections is largely focused on surgical technique, antibiotic prophylaxis, infection prevention, and traditional maternal factors. In the context of cesarean outcomes, further exploration of endocrine markers of postoperative recovery is relatively understudied. Serial assessment of serum β-hCG levels may provide clinically relevant data about the time, quality, and profile of abdominal wound healing following cesarean section. The present study compares postoperative serum β-hCG levels among women who have cesarean delivery and investigates the relationship between the pattern of β-hCG decrease and the outcome of abdominal wound healing.

Objectives of the study

The primary objective was to evaluate the association between serial postoperative serum β-hCG trends and abdominal wound-healing status after cesarean section. The secondary objectives were to characterize the pattern of β-hCG decline across predefined postoperative time points and to assess the discriminatory ability of postoperative day (POD) 7 serum β-hCG for distinguishing between healthy and unhealthy wound outcomes using receiver operating characteristic (ROC) analysis.

## Materials and methods

Study design and setting

This was an observational analytical study conducted in a tertiary care teaching hospital in the Department of Obstetrics and Gynecology. Women who underwent uncomplicated lower-segment cesarean section (LSCS), either electively or as an emergency procedure, during the defined study period were recruited. This study aimed to compare postoperative serum β-hCG levels in different groups of women after cesarean section and to examine the correlation between postoperative serum β-hCG trends and abdominal wound healing after cesarean section. Standardized perioperative documentation, as well as sequential biochemical testing and a structured postoperative wound assessment during follow-up, were possible in the hospital setting. β-hCG levels were measured from the beginning of the study until POD 21 to assess the postoperative course of β-hCG and its correlation with wound healing.

Study population and eligibility criteria

Women undergoing LSCS who satisfied the eligibility criteria were screened during the study period. Wound status was not assigned at enrollment. Participants were clinically assessed on POD 3 and subsequently classified according to wound outcome. A total of 218 women with signs of wound infection were included as cases, while 218 women with healthy wounds were included as controls, resulting in a final analytical sample of 436 participants. Eligible participants had a singleton pregnancy, delivered a live-born infant by cesarean section at the study institution, provided written informed consent for study participation and serial blood sampling, and were available for postoperative follow-up through day 21. Women with known diabetes mellitus, an immunocompromised state, chronic systemic corticosteroid therapy, a known connective tissue disorder, severe anemia requiring massive blood transfusion, postpartum hemorrhage requiring relaparotomy, refusal to provide consent, or inability to complete postoperative follow-up were excluded. Both elective and emergency cesarean sections were included.

Potential confounding was addressed partly through the eligibility criteria and partly through comparative assessment of measured maternal and obstetric characteristics. Exclusion of women with known diabetes mellitus and other major conditions associated with impaired wound healing was intended to reduce the influence of established clinical confounders. Maternal BMI, gestational age at delivery, and elective versus emergency cesarean section status were recorded and compared between the healthy and unhealthy wound groups. No statistically significant between-group associations were observed for these variables. Detailed data on duration of rupture of membranes, chorioamnionitis, operative duration, suture type, and subcutaneous tissue closure were not sufficiently complete for reliable multivariable adjustment. These factors, together with maternal renal clearance, placental mass, and other unmeasured biological and perioperative characteristics, were therefore considered potential sources of residual confounding. The detailed inclusion and exclusion criteria are presented in Table 1.

**Table 1:** Eligibility criteria for study participation. LSCS: lower-segment cesarean section; POD: postoperative day

Eligibility category	Criterion
Inclusion criterion	Women who underwent LSCS
	Singleton pregnancy
	Delivery of a live-born infant
	Provision of written informed consent for study participation and serial blood sampling
	Availability for postoperative follow-up through POD 21
Exclusion criterion	Known diabetes mellitus
	Immunocompromised state
	Chronic systemic corticosteroid therapy
	Known connective tissue disorder
	Severe anemia requiring massive blood transfusion
	Postpartum hemorrhage requiring relaparotomy
	Refusal to provide consent or inability to complete postoperative follow-up

These exclusion criteria were applied to minimize the influence of established clinical factors that could independently impair wound healing and to allow clearer evaluation of the discriminatory performance of β-hCG in relation to wound outcome. However, these exclusions limit the generalizability of the findings, which should not be directly extrapolated to women with these comorbidities or major postpartum complications.

Sample size calculation

The primary sample size calculation was based on a two-group comparison of independent continuous outcomes, consistent with the planned comparison between women with unhealthy and healthy wounds. For equal allocation between the two groups, the required sample size was calculated using the following formula:

$n = \frac{2\sigma^2(Z_{\alpha/2}+Z_{\beta})^2}{\Delta^2}$

where n represents the required number of participants per group, σ represents the assumed standard deviation, Δ represents the expected between-group difference, Zα/2 corresponds to the two-sided type I error, and Zβ corresponds to the desired statistical power. Assuming a standard deviation of 2.4, an expected between-group difference of 0.65, a two-sided alpha level of 0.05 (Zα/2 = 1.96), and 80% power (Zβ = 0.842), the standardized effect size was approximately 0.271. Substituting these values yielded:

$n = \frac{2(2.4)^2(1.96+0.842)^2}{(0.65)^2} = 213.77$

Accordingly, a minimum of 214 participants per group, or 428 participants overall, was required. The final analytical sample included 436 women, comprising 218 women with unhealthy wounds and 218 women with healthy wounds, thereby exceeding the prespecified minimum requirement. The previously considered prevalence-based estimate derived from the reported incidence of post-cesarean surgical-site infection was used only as a preliminary estimate of the potentially available study population and was not used as the primary inferential sample size justification.

Data collection and baseline assessment

Data on sociodemographic and obstetric parameters were obtained on a predesigned and pretested proforma. Assessed variables included age, BMI, socioeconomic status, gravida, parity, abortion history, live births, number of antenatal visits, diet history, drug intake, associated comorbidities, and previous surgical history. Hematological status was determined by obtaining the preoperative hemoglobin level from routine laboratory investigations. Details were recorded systematically, including the type of LSCS (elective or emergency), indication for LSCS, presence and severity of intraoperative complications, and requirement for blood transfusion. A uniform system was used to collect clinical and perioperative data.

Serum β-hCG measurement

Serial serum β-hCG estimation was performed for each participant at nine predefined time points: pre-LSCS, immediately postoperatively, and on PODs 2, 3, 4, 5, 7, 14, and 21. Blood samples were collected under aseptic conditions and processed in the hospital laboratory using a standardized immunoassay method. Absolute serum β-hCG concentrations were evaluated at each predefined time point, together with the pattern of postoperative decline. Because wound status was determined on POD3, subsequent β-hCG measurements, including POD7 values, were assessed for their association with and discriminatory ability between previously classified wound-outcome groups rather than as prospective predictors.

Wound assessment and outcome measures

Abdominal wounds were clinically examined by the same doctor on PODs 2, 3, 4, 5, 7, 14, and 21 using Centers for Disease Control and Prevention/National Healthcare Safety Network criteria for surgical-site infection [18]. Wound outcomes were classified into two categories: healthy and unhealthy. The unhealthy-wound category included superficial surgical-site infection, deep incisional surgical-site infection, and organ/space surgical-site infection [18]. Wound status used for case-control classification was determined on POD3. The primary outcome was the association between serial serum β-hCG decline and wound-healing status. Secondary outcomes included the postoperative pattern of β-hCG decline and the discriminatory ability of POD7 β-hCG to distinguish between healthy and unhealthy wound outcomes.

Statistical analysis

Data were analyzed using IBM SPSS Statistics for Windows, version 23.0 (released 2015; IBM Corp., Armonk, NY, USA). The study was powered primarily for the independent two-group comparison, with 218 participants in each group exceeding the prespecified minimum requirement of 214 participants per group. Categorical variables were summarized as frequencies and percentages, and continuous variables were expressed as mean ± standard deviation. Independent-samples t-tests were used to compare β-hCG concentrations between healthy and unhealthy wound groups at each predefined postoperative time point, while categorical variables were analyzed using the chi-square test. Maternal BMI, gestational age at delivery, and elective versus emergency cesarean section status were specifically examined as measured potential confounders and showed no statistically significant between-group associations. Women with known diabetes mellitus and several other major conditions associated with impaired wound healing were excluded at the design stage. Serial β-hCG measurements were evaluated as separate between-group comparisons at each predefined time point; no longitudinal repeated-measures model was applied. ROC analysis was performed as a secondary analysis to assess the discriminatory ability of POD 7 serum β-hCG for wound outcome, and the optimal cutoff was determined using the Youden index on the same study dataset. Multivariable adjustment for duration of rupture of membranes, chorioamnionitis, operative duration, suture type, and subcutaneous tissue closure could not be performed because sufficiently complete analyzable data for these variables were unavailable. Consequently, residual confounding by these maternal and perioperative factors cannot be excluded, and β-hCG was not interpreted as an independent predictor of wound outcome. A two-sided p-value <0.05 was considered statistically significant.

## Results

Demographic and obstetric characteristics

No statistically significant associations were observed between wound-healing outcome and age group, BMI category, gravida, parity, abortion history, live-birth history, previous surgery, gestational age, or type of cesarean section. Among the measured potential confounders, BMI category, gestational age at delivery, and elective versus emergency cesarean section status were not significantly associated with wound-healing outcome. BMI showed χ² = 3.041, p = 0.385, and Cramér’s V = 0.084; gestational age showed χ² = 2.563, p = 0.464, and Cramér’s V = 0.077; and cesarean type showed χ² = 0.270, p = 0.603, and Cramér’s V = 0.025. These findings indicated negligible-to-small unadjusted associations with wound outcome. Table 2 presents the demographic and obstetric characteristics of the 436 participants.

**Table 2:** Baseline demographic and obstetric characteristics. G: gravida, LSCS: lower-segment cesarean section

Variable	Category	Total, n (%)	Unhealthy wound, n (%)	Healthy wound, n (%)	χ²	df	p-value	Cramér’s V
Age group	<19 years	11 (2.5%)	6 (2.8%)	5 (2.3%)	0.419	2	0.811	0.031
	19-30 years	306 (70.2%)	150 (68.8%)	156 (71.6%)				
	31-38 years	119 (27.3%)	62 (28.4%)	57 (26.1%)				
BMI category	Underweight (<18.5 kg/m²)	9 (2.1%)	5 (2.3%)	4 (1.8%)	3.041	3	0.385	0.084
	Normal (18.5-22.9 kg/m²)	331 (75.9%)	158 (72.5%)	173 (79.4%)				
	Overweight (23.0-24.9 kg/m²)	80 (18.3%)	45 (20.6%)	35 (16.1%)				
	Pre-obese/obese (≥25.0 kg/m²)	16 (3.7%)	10 (4.6%)	6 (2.8%)				
Gravida category	Primigravida (G1)	168 (38.5%)	83 (38.1%)	85 (39.0%)	0.039	2	0.981	0.009
	Multigravida (G2-G4)	131 (30.0%)	66 (30.3%)	65 (29.8%)				
	Grand multigravida (≥G5)	137 (31.5%)	69 (31.7%)	68 (31.2%)				
Parity category	Nulliparous	203 (46.6%)	100 (45.9%)	103 (47.2%)	0.260	2	0.878	0.024
	Para 1	149 (34.2%)	77 (35.3%)	72 (33.0%)				
	Para ≥2	84 (19.3%)	41 (18.8%)	43 (19.7%)				
Abortion history	None	324 (74.3%)	163 (74.8%)	161 (73.9%)	3.637	2	0.162	0.091
	One	86 (19.7%)	38 (17.4%)	48 (22.0%)				
	≥2	26 (6.0%)	17 (7.8%)	9 (4.1%)				
Live-birth history	No live birth	190 (43.6%)	91 (41.7%)	99 (45.4%)	0.762	2	0.683	0.042
	One live birth	201 (46.1%)	105 (48.2%)	96 (44.0%)				
	≥2 live births	45 (10.3%)	22 (10.1%)	23 (10.6%)				
Previous surgery	No	374 (85.8%)	192 (88.1%)	182 (83.5%)	1.880	1	0.17	0.066
	Yes	62 (14.2%)	26 (11.9%)	36 (16.5%)				
Gestational age	<34 weeks	23 (5.3%)	14 (6.4%)	9 (4.1%)	2.563	3	0.464	0.077
	34-36 + 6 weeks	135 (31.0%)	62 (28.4%)	73 (33.5%)				
	37-40 + 6 weeks	161 (36.9%)	85 (39.0%)	76 (34.9%)				
	≥41 weeks	117 (26.8%)	57 (26.1%)	60 (27.5%)				
Type of LSCS	Elective	133 (30.5%)	69 (31.7%)	64 (29.4%)	0.270	1	0.603	0.025
	Emergency	303 (69.5%)	149 (68.3%)	154 (70.6%)				

Serial postoperative serum β-hCG trend

A progressive decrease in serum β-hCG was observed at each postoperative measurement. The preoperative mean level was 15,881.99 IU/L, and the concentration decreased slightly immediately after surgery to 15,168.07 IU/L. A greater decrease was observed on PODs 2, 3, 4, 5, and 7. Additional measurements showed that the concentration decreased to 525.57 IU/L on POD 14 and 143.13 IU/L on POD 21. Median and maximum values also decreased consistently, reflecting a postpartum clearance pattern following cesarean delivery. Table 3 shows a continuous decline in serum β-hCG from the preoperative period to POD 21, with a marked reduction after POD 2.

**Table 3:** Serial serum β-hCG concentrations across the postoperative timeline. β-hCG: beta-human chorionic gonadotropin; POD: postoperative day

β-hCG time point (IU/L)	Mean	SD	Median	Minimum	Maximum
Preoperative	15,881.99	5406.62	16,491.50	6003.00	24,933.00
Immediate postoperative	15,168.07	5187.96	15,744.00	5541.00	24,609.00
POD 2	8513.28	3332.25	8438.50	2251.00	18,112.00
POD 3	6476.80	2910.43	6130.00	1357.00	16,342.00
POD 4	4971.96	2571.60	4552.50	777.00	14,319.00
POD 5	3847.51	2275.88	3406.50	474.00	12,358.00
POD 7	2366.76	1775.25	1912.00	167.00	9546.00
POD 14	525.57	677.69	254.50	5.00	3620.00
POD 21	143.13	247.16	33.00	1.00	1435.00

Comparison of β-hCG levels by wound-healing status

There was no statistically significant difference in preoperative or immediate postoperative serum β-hCG levels between women with unhealthy and healthy wounds. From POD 2 onward, women with unhealthy wounds had significantly higher mean β-hCG concentrations than those with healthy wounds, and the between-group difference remained evident on PODs 3, 4, 5, 7, 14, and 21. All between-group comparisons from POD 2 through POD 21 were statistically significant (p < 0.001). These findings indicate an association between persistently higher postoperative β-hCG concentrations and unhealthy wound outcomes. As measurements were analyzed as separate between-group comparisons at predefined time points rather than with a longitudinal repeated-measures model, the findings should not be interpreted as demonstrating an independently modeled difference in β-hCG trajectories over time. Table 4 presents the β-hCG concentrations according to wound-healing status at each assessed time point.

**Table 4:** β-hCG levels by wound outcome. β-hCG: beta-human chorionic gonadotropin; POD: postoperative day

β-hCG time point (IU/L)	Unhealthy wound (n = 218)	Healthy wound (n = 218)	t-value	p-value
Preoperative	15,682.91 ± 5170.69	16,081.08 ± 5637.56	0.769	0.443
Immediate postoperative	14,968.11 ± 4961.81	15,368.02 ± 5408.71	0.804	0.422
POD 2	9730.50 ± 3401.44	7296.05 ± 2777.09	8.186	<0.001
POD 3	7887.83 ± 2926.71	5065.77 ± 2101.00	11.565	<0.001
POD 4	6403.29 ± 2548.85	3540.63 ± 1625.96	13.98	<0.001
POD 5	5207.96 ± 2248.48	2487.06 ± 1268.38	15.562	<0.001
POD 7	3488.66 ± 1787.84	1244.86 ± 769.57	17.021	<0.001
POD 14	926.42 ± 762.97	124.72 ± 125.26	15.309	<0.001
POD 21	271.43 ± 298.31	14.83 ± 20.02	12.671	<0.001

ROC analysis for POD7 β-hCG

A ROC analysis was used to assess the ability of serum β-hCG on POD 7 to discriminate between healthy and unhealthy wound outcomes. The area under the curve was 0.901, indicating good discriminatory ability. An optimal threshold of 1,884 IU/L was determined using the Youden index, with a sensitivity of 82.6% and specificity of 81.2%, indicating a good balance of diagnostic performance. These results suggest that the β-hCG level on POD 7 can differentiate women with unhealthy post-cesarean wound outcomes from those with healthy wound healing. The serum β-hCG level on POD 7 had good discriminatory power for unhealthy outcomes (Table 5).

**Table 5:** POD 7 β-hCG for discriminating wound outcome. β-hCG: beta-human chorionic gonadotropin; POD: postoperative day; ROC: receiver operating characteristic

ROC parameter	Value
Area under the curve	0.901
Optimal cutoff by Youden index	1884 IU/L
Sensitivity at cutoff	0.826
Specificity at cutoff	0.812

Figure 1 demonstrates the discriminatory ability of POD 7 serum β-hCG between healthy and unhealthy wound outcomes, with an area under the curve of 0.901 (95% CI: 0.874-0.929).

**Figure 1:**
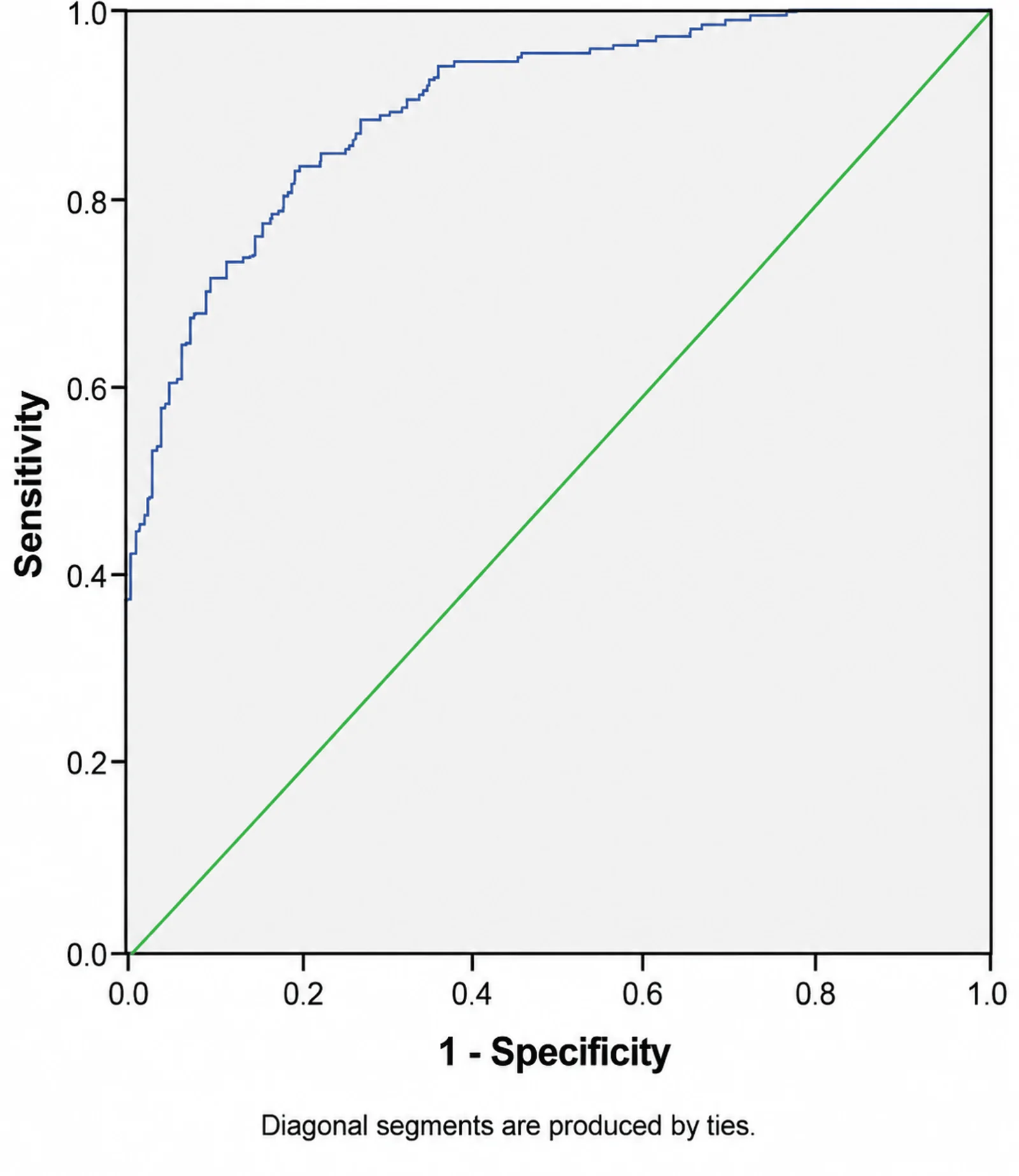
ROC curve for POD7 β-hCG predicting unhealthy wound outcome. The blue line represents the ROC curve for POD 7 serum β-hCG, and the green diagonal line represents the reference line corresponding to no discriminatory ability (AUC = 0.50). The observed AUC was 0.901. AUC, area under the curve; β-hCG: beta-human chorionic gonadotropin; POD: postoperative day; ROC: receiver operating characteristic

## Discussion

The present results suggest that the study population was clinically relevant, with the majority of women in the reproductive age group, a predominantly normal BMI, and a greater number of emergency cesarean sections. This demographic and obstetric distribution is crucial to the interpretation of postoperative wound outcomes, as emergency operative delivery may be performed under less ideal clinical circumstances than elective delivery. Primigravida, multigravida, and grand multigravida women were also represented, providing a broad obstetric range for assessing the trend of serum β-hCG levels. In previous cohorts of cesarean deliveries, similar age distribution, parity patterns, gestational age distribution, and predominance of emergency cesarean deliveries were observed [16,19]. Postoperative serum β-hCG levels consistently decreased from the preoperative time point to 21 days after surgery. This reduction was greatest in the early postoperative period, especially between PODs 2 and 7, followed by a progressive decrease later in the postoperative period. This is similar to the physiological postsurgical decrease in the production of placental hormones following delivery. The significant decrease in the range of values at later stages indicates progressive normalization of serum β-hCG following cesarean section. The same studies that evaluated the resolution of β-hCG following pregnancy-related uterine interventions also reported a progressive decrease over time after surgery [14].

There was no significant difference in preoperative or immediate postoperative β-hCG levels between the healthy and unhealthy wound groups, indicating comparable baseline hormone levels before the groups diverged clinically. From POD 2 onward, women with unhealthy wounds showed persistently higher β-hCG levels, suggesting an association between slower β-hCG clearance and impaired wound healing. The increasing separation between groups on PODs 3, 4, 5, and 7 may be more clinically informative than a single perioperative measurement. Previous literature has described β-hCG-related effects involving immune modulation, vascular activity, inflammatory regulation, and tissue-remodeling pathways [12,20]. These mechanisms may provide biologically plausible explanations for the observed association; however, they were not directly evaluated in the present study. Delayed β-hCG decline may therefore reflect altered postpartum endocrine or systemic recovery, although this remains hypothetical. Previous studies have similarly reported variable β-hCG clearance patterns following pregnancy-related events and interventions [21].

There were statistically significant differences between the wound groups at all time points beginning on POD 2 and continuing through POD 21. This prolonged separation further supports the clinical relevance of analyzing β-hCG as a postoperative marker. Persistently elevated β-hCG levels were associated with unhealthy wound outcomes, while a more rapid decline in β-hCG was associated with healthy wound healing. Previous studies have demonstrated that β-hCG resolution varies in different clinical situations and may differ by day or even week following pregnancy-related events [19,21]. The present results highlight this concept and support the study of β-hCG dynamics in relation to the healing status of the abdominal skin after cesarean section. The sensitivity and specificity of serum β-hCG collected on POD 7 were good for identifying adverse wound-healing outcomes in the ROC analysis. An area under the curve of 0.901 suggests good discriminatory ability, and an optimal threshold of 1,884 IU/L provides a balance of sensitivity and specificity. This finding suggests that POD 7 is a clinically informative time point, as day 7 may be when wound vulnerability and the risk of early dehiscence become clinically apparent [10]. A β-hCG level exceeding this cutoff could help identify women who require more careful wound monitoring, postoperative education, and clinical follow-up.

Clinical implications of these findings include the possibility of using serial serum β-hCG trends as a supplement to routine post-cesarean wound assessment. Discharge, infection, gaping, delayed healing, and dehiscence are all clinical signs that require clinical examination. Before clinical deterioration of wounds is observed, biochemical monitoring may provide additional risk stratification. POD7 β-hCG is of particular interest because it may have clinical utility. Incorporating β-hCG kinetics with conventional clinical assessment could further help identify women with poor wound healing after cesarean section. Furthermore, the findings of the present study corroborate a more general concept that recovery after cesarean section should be considered not only in terms of surgical or infectious parameters but also in terms of overall physiological recovery. Postpartum endocrine transition may provide further insights into maternal biological recovery. The focus of current obstetric literature on cesarean wound morbidity has been on operative indications, surgical technique, antibiotic prophylaxis, infection prevention, and traditional maternal risk factors [13,22]. Based on the findings of serial β-hCG evaluation, it may be concluded that endocrine biomarkers can serve as an adjunct to postoperative care of obstetric and gynecologic patients, especially in hospitals with high cesarean rates and early discharge.

Limitations and future directions

This single-center observational study has several limitations. Although women with major conditions known to impair wound healing, including known diabetes mellitus, were excluded, residual confounding remains possible. Maternal BMI, gestational age, and elective versus emergency cesarean section status showed no significant unadjusted association with wound outcome, but other important factors, including duration of rupture of membranes, chorioamnionitis, operative duration, suture type, subcutaneous tissue closure, maternal renal function, placental mass, nutritional status, hemoglobin level, antibiotic exposure, transfusion, wound care, and operative technique, were not sufficiently complete for reliable multivariable adjustment. The association between postoperative β-hCG and wound outcome therefore cannot be interpreted as independent of all maternal, obstetric, and surgical confounders. Clinical wound assessment may be observer-dependent, and wound groups were defined after POD 3 assessment rather than by prospective allocation. Serial β-hCG values were compared at individual time points without longitudinal repeated-measures modeling. The POD 7 cutoff was derived and evaluated in the same dataset without independent validation, so its discriminatory performance may be optimistic and should not be considered an established predictive threshold.

Future multicenter studies should prospectively collect complete maternal, obstetric, and operative data, apply multivariable and longitudinal modeling, and externally validate the POD 7 β-hCG cutoff using standardized wound-assessment methods and longer follow-up.

## Conclusions

The study identified an association between slower postpartum serum β-hCG decline and unhealthy abdominal wound outcomes following cesarean section. Women with unhealthy wounds demonstrated persistently higher β-hCG levels from POD 2 onward. POD 7 β-hCG showed good discriminatory ability between healthy and unhealthy wound groups; however, the observational design, absence of multivariable adjustment, and lack of independent validation preclude conclusions regarding causality or independent predictive performance. Serial β-hCG assessment may therefore have potential as an adjunctive marker of postoperative wound status, but its clinical utility and the proposed POD7 cutoff require confirmation in larger, multicenter studies with appropriate adjustment for confounding and external validation.
